# Severe Acute Hepatitis B Treated With Entecavir.

**DOI:** 10.4084/MJHID.2011.010

**Published:** 2011-03-15

**Authors:** Giuseppe Vittorio Luigi De Socio, Alessio Sgrelli, Andrea Tosti, Franco Baldelli

**Affiliations:** Department of Infectious Diseases “Santa Maria della Misericordia” Hospital, University of Perugia, Perugia Italy

## Abstract

Hepatitis B virus (HBV) infection constitutes a serious global health problem. Nowadays there are divergent data regarding the use of antiviral drugs to treat acute hepatitis B. We present here a case of a 62-year-old man affected by severe acute hepatitis B with progressive worsening of clinical and hepatic function. The patient was treated with entecavir without critical side effects. We observed rapid clinical and laboratory improvements and the disappearance of hepatitis B surface antigen (HBsAg). The treatment with entecavir was protracted until 17^th^ week when the antibody anti-HBs appeared. Entecavir should be carefully considered for the treatment of severe acute hepatitis B cases.

## Introduction:

Acute hepatitis B viral (HBV) infection can sometimes take a severe course, leading to liver failure due to hepatic necrosis with a death rate of 80%.^1^–^2^ Emergency liver transplantation is often the only therapeutic option available.^3^ There are many drugs approved for the treatment of chronic hepatitis B, that inhibit the replication of the HBV with a low toxicity and a good resistance profile such lamivudine, adefovir, tenofovir, entecavir and telbivudine. Given their safety, these drugs may represent a reasonable therapeutic option also in severe acute hepatitis B.^3^–^6^ The new guidelines endorsed by America Association for the study of liver diseases (AASLD) recommend to treat symptomatic acute hepatitis in patients affected with fulminant hepatitis and by severe acute hepatitis B and suggesting the use of lamivudine or telbivudine when the anticipated duration of treatment is short; otherwise, entecavir is preferred.^7^

Patients are considered to have severe acute viral hepatitis B if they fulfill 2 of 3 criteria: (1) hepatic encephalopathy; (2) serum bilirubin level > 10.0mg/dL; and (3) international normalized ratio (INR) >1.6.^7^–^9^

## Case Report:

A 62-year-old Italian man was admitted to the hospital because of jaundice, fever, fatigue, loss of appetite and dark urine. His history was negative for any major illness, nor alcohol or drugs abuse. He referred unprotect sexual contact two months before. At hospital admission the examination showed a frank jaundice and hepatomegaly. No splenomegaly, nor flapping tremor, nor sleep or digestive disorders were observed. The laboratory exams showed alanine aminotransferase (ALT) 2304 mU/mL, aspartate aminotransferase (AST) 1595mU/mL, gamma glutamil trans peptidase (γGT) 389 mU/mL, direct bilirubin level 6.64 mg/dL, total bilirubin level 9.53 mg/dL, prothrombin time activity percentage (PT) 64%, INR 1.30, Glycaemia level 98mg/dL, ammonia 88 mg/dL, total cholesterol 117 mg/dL, HDL-cholesterol 13 mg/dL. Hepatitis B markers were hepatitis B surface antigen (HBsAg) positive, quantitative HBsAg >250.00 UI/ml, antibodies anti surface antigen (anti-HBs) were negative, antibodies to e antigen (anti-HBe) and antibodies to core antigen (anti-HBc) IgM both positive, IgM index (sample/cut-off ratio) 25.98, HBV DNA 534258 UI/mL. The other hepatitis virus markers (HAV, HCV, HDV, EBV, CMV) were all negative for acute infection. His serologic test was diagnostic for acute hepatitis B and the diagnosis was strongly supported by the finding of high IgM core index.^10^ To correct the INR we administered 10 mg per day of phytomenadione for three days without improvement. The patient showed a progressive worsening of the clinical conditions with slight mental confusion, sleep-wake rhythm alteration, reduction of alertness, anorexia, fever, marked asthenia, jaundice worsening, spleen enlargement (one cm from the costal arch) and increasing hepatomegaly.

The laboratory examination showed a worsening condition: INR 1.49, ammonia 105 mg/dL, total cholesterol 94 mg/dL, HDL cholesterol 12 mg/dL AST 2237 mU/mL, ALT 3034 mU/mL, total bilirubin level 19.36 mg/dL. Electroencephalogram (EEG) showed anomalies of the background activities, while the abdominal echography confirmed the presence of hepato-splenomegaly and marked the presence of small ascitic fluid that wasn’t detected when the patient was hospitalized.

**Figure 1:**
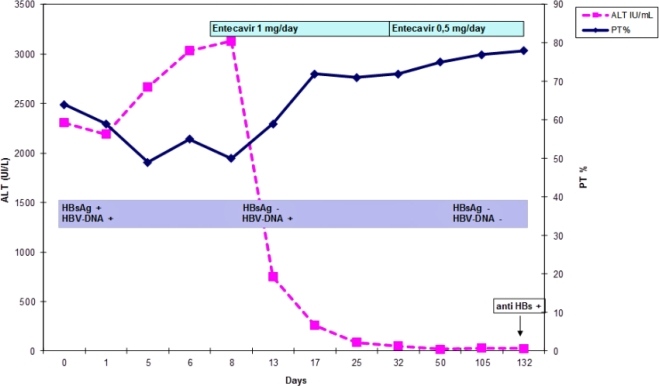
Biochemical and viral parameters at admission and subsequent controls. Alanine aminotransferase (ALT) UI/mL, prothrombin time activity percentage (PT), hepatitis B antigen (HBsAg) UI/mL, antibody antiHBs (anti HBs), Hepatitis B DNA (HBV-DNA) UI/mL.

At this stage, the patient presented criteria of severe acute hepatitis B, thus together with general supportive therapies as glucose solutions iv and lactulose solutions po, we started “off label” treatment with entecavir 1 mg daily. The patient was informed that the drug was not approved for this indication and he gave the consent for the treatment. After three days of antiviral treatment the patient was stably apiretic, while after ten days anorexia, mental confusion, sleep-wake rhythm alteration, asthenia disappeared, jaundice and hepatomegaly were reduced. Ten days after the start of entecavir treatment, the laboratory exams confirmed the improvement of hepatic function: INR 1.21, ammonia 79 mg/dL, total cholesterol level 159 mg/dL, AST 72 mU/mL, ALT 261 mU/mL, total bilirubin level 13.29 mg/dL (Figure 1). Hepatitis markers revealed: HBsAg and quantitative HBsAg both negative and HBV DNA 65 UI/mL (Figure 1).

The patient was discharged after 15 days from the beginning of the antiviral treatment. At follow-up after three weeks from starting entecavir, ascitic effusions disappeared, the liver and spleen dimension and coagulation parameters were all normalized. Therapy was continued with entecavir 1 mg day for five weeks, the dosage was decreased to 0.5 mg as the patient experienced a growth of hair loss. HBV DNA wasn’t detected after six weeks (figure 1). We continued the treatment with entecavir until the 17th week, when the anti-HBs appeared (15 UI/mL).

## Discussion:

We observed rapid improvement of a severe acute hepatitis B treated with entecavir, in accordance with previous reports.^3^,^6^,^11^ In medical literature there are divergent data regarding the use of antiviral drugs to treat acute severe hepatitis B. For instance, lamivudine has proved no better than placebo,^8^ while others showed a reduction of mortality rate.^4^,^6^ Theoretically lamivudine or telbivudine would be a reasonable choice given their safety and rapidity of action. Unfortunately HBV-resistance to lamivudine is increasingly reported. Monotherapy with entecavir, a new generation drug, showed encouraging preliminary results in patients affected by acute severe hepatitis B.^3^,^6^,^11^ Furthermore clinical trials of long-term entecavir treatment of chronic hepatitis B have shown a very low rate of resistance to this drug compared with other antiviral. So in severe acute hepatitis B, this drug seems to be a good reasonable choice when resistance profile is high or the patient could be considered at risk of infection by resistant virus.^7^ Tenofovir and adefovir may not be optimal because of their potential for nephrotoxicity.^7^ We didn’t know the patient’s viral HBV resistance profile, so for safety and according to Jochum et al^11^ we used entecavir at the dose of 1 mg for day considering the approved dose for lamivudine resistant patients.^7^

The main concerns about the use of anti-HBV drugs during the acute phase are principally linked to two factors: first, the antiviral therapy is generally not necessary because more than 95% of acute infections solve spontaneously.^7^ Second, early antiviral therapy theoretically may inhibit the production of neutralizing antibody in the early phase of the disease and delays the appearance of anti-HBs. In a recent study lamivudine used in acute hepatitis B has been reported to determine a reduced anti-HBs seroconversion as compared to patients not receiving the antiviral,^4^ even if other studies did not observe significant differences.^8^ Usually anti-HBs appear after one - two months from HBsAg disappearance, although in some patients not being treated with antiviral drugs, antibodies could not appear in weeks or even months.^12^ In the reported case, antibodies anti-HBs appeared after about four months from the disappearance of the HBsAg. The duration of antiviral treatment is not established; we stopped entecavir at seroconversion to anti-HBs. However, this issue is still controversial: EASL guidelines recommend therapy to be continued for at least 3 months after anti-HBs appearance,^13^ while AASLD ones until HBsAg clearance is confirmed.^7^ Further studies are necessary to support the appropriateness and the duration of entecavir treatment in cases of acute severe hepatitis B. Meanwhile the possible use of this drug should be considered for selected patients with acute hepatitis B.
